# A Monte Carlo approach for scattering correction towards quantitative neutron imaging of polycrystals

**DOI:** 10.1107/S1600576718001607

**Published:** 2018-03-01

**Authors:** M. Raventós, E. H. Lehmann, M. Boin, M. Morgano, J. Hovind, R. Harti, J. Valsecchi, A. Kaestner, C. Carminati, P. Boillat, P. Trtik, F. Schmid, M. Siegwart, D. Mannes, M. Strobl, C. Grünzweig

**Affiliations:** a Laboratory for Neutron Scattering and Imaging, Villigen, Switzerland; b University of Geneva, Geneva, Switzerland; c Helmholtz Zentrum Berlin für Materialien und Energie, Berlin, Germany

**Keywords:** neutron imaging, quantification, neutron scattering, Monte Carlo methods

## Abstract

The development of neutron imaging from a qualitative inspection tool towards a quantitative technique in materials science has increased the requirements for accuracy significantly. Quantifying the thickness or the density of polycrystalline samples with high accuracy using neutron imaging has two main problems: (i) the scattering from the sample creates artefacts on the image and (ii) there is a lack of specific reference attenuation coefficients. This work presents experimental and simulation results to explain and approach these problems. Firstly, a series of neutron radiography and tomography experiments of iron, copper and vanadium are performed and serve as a reference. These materials were selected because they attenuate neutrons mainly through coherent (Fe and Cu) and incoherent (V) scattering. Secondly, an *ad hoc* Monte Carlo model was developed, based on beamline, sample and detector parameters, in order to simulate experiments, understand the physics involved and interpret the experimental data. The model, developed in the *McStas* framework, uses *a priori* information about the sample geometry and crystalline structure, as well as beamline settings, such as spectrum, geometry and detector type. The validity of the simulations is then verified with experimental results for the two problems that motivated this work: (i) the scattering distribution in transmission imaging and (ii) the calculated attenuation coefficients.

## Abstract 2

This article describes the development and application of a Monte Carlo tool to improve the quantification capabilities of neutron imaging applied to polycrystals. The combination of modelling and experimentation gives a better understanding of how scattering coming from polycrystalline samples affects neutron imaging experiments.

## 1. Introduction and motivation   

Neutron imaging is a well established technique for nondestructive two-, three- and four-dimensional evaluation of samples (*e.g.* Anderson *et al.*, 2009 ▸; Strobl *et al.*, 2009 ▸; Kaestner, Mnch *et al.*, 2011 ▸). Thermal and cold neutrons have wavelengths of the order of inter-atomic lattice distances, which are suitable for diffraction applications and combinations of imaging and diffraction (Santisteban *et al.*, 2002 ▸; Peetermans *et al.*, 2014 ▸; Woracek *et al.*, 2017 ▸; Cereser *et al.*, 2017 ▸). As for standard neutron transmission imaging, identification of materials with different attenuation is based on the Beer–Lambert law: 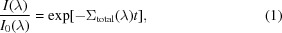
where 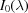 is the initial intensity of the beam, 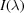 is the transmitted intensity, 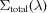 is the attenuation coefficient of the material and *t* is the thickness of the sample in the beam direction. The attenuation coefficient is defined as 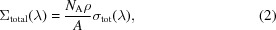
where 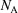 is the Avogadro constant, ρ is the mass density of the material, *A* is the atomic mass and 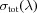 is the wavelength-dependent total microscopic cross section.

To be able to quantify the attenuation of a sample, we approach two unsolved questions: how to avoid scattered neutrons adding intensity to the transmission images and how to obtain adequate cross section values.

In the first place, classic neutron imaging considers the transmission through the sample and thus measures the attenuation coefficient [equation (2)] as a line integral through the probed material. Neutrons originating from coherent, incoherent, elastic and inelastic scattering are considered as absorbed. However, a portion of the neutrons scattered close to the forward direction are bound to be captured by the transmission detector, thus distorting the results with respect to the Beer–Lambert law. This poses a challenge for quantification, which has been previously tackled using scattering correction tools (Kardjilov *et al.*, 2005 ▸; Hassanein *et al.*, 2005 ▸), calibration (Pekula *et al.*, 2005 ▸), wavelength selection (Treimer *et al.*, 2006 ▸) and collimators (Tremsin *et al.*, 2011 ▸). Scattering correction tools have been used successfully for water quantification, although detailed information on the sample geometry and composition is required, and the choice of correction parameters may introduce a bias in the measurement. Calibration of the sample gives an estimate of the expected attenuation of a sample but does not solve the scattering problem. Selecting wavelengths longer than the Bragg cut-off of the sample avoids only Bragg scattering contributions and it usually requires selecting only the coldest neutrons of the beam, leading to a trade-off between quantitativeness and neutron intensity, and hence experimental time. Finally, collimators can be effective for scattering removal, but they have to be placed between the detector and sample and have an impact on the beam collimation and geometry.

In the second place, the expected cross section value of a sample is often not trivial to compute. The absorption and incoherent cross sections depend on the target nucleus, while the coherent scattering cross section depends also on the crystal structure and microstructure of the sample material. Unfortunately, wavelength-dependent cross section data in the cold region are rare in nuclear databases. These databases were created to serve as reference for criticality considerations and operation of power plants, in which the cold region is irrelevant (Otuka *et al.*, 2014 ▸). Therefore, reference cross section data which are precise and representative of the wavelength spectrum of various available neutron imaging beamlines are required.

**Figure 1:**
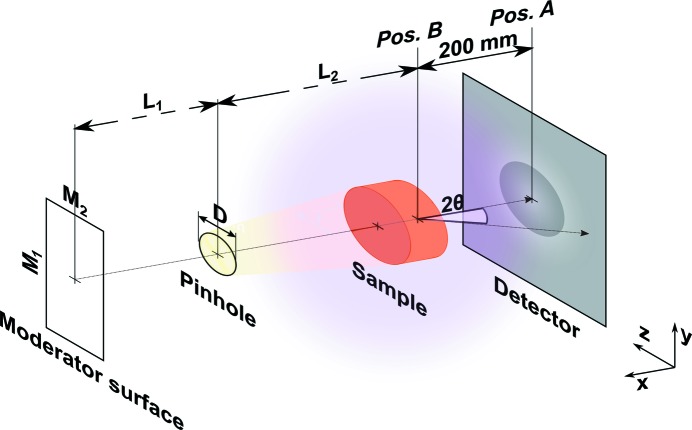
Schematic layout of a neutron imaging beamline. The depicted components are key to defining the input parameters of the Monte Carlo model. Neutrons travel from the moderator surface (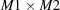) through the pinhole with diameter *D. L*_1_ is the distance between the moderator and the pinhole and *L*_2_ is the distance between the pinhole and the sample. The yellow shadow represents the slightly divergent neutron beam which illuminates the sample and the pink shadow the neutrons scattered from the sample. A sample in position A (Pos. A) is in close contact with the detector, while a sample in position B (Pos. B) is at 200 mm distance from it. As an example, the dimensions for the NEUTRA beamline are (in mm) 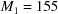, 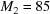, 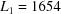, 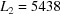, 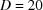.

Imaging beamlines at PSI are represented in the thermal, thermal–cold and cold ranges by NEUTRA (Lehmann *et al.*, 2001 ▸), ICON (Kaestner, Hartmann *et al.*, 2011 ▸) and BOA (Morgano *et al.*, 2014 ▸). Fig. 1 ▸ shows the scheme of a typical neutron imaging beamline.

With a beamline layout like the one described above, the maximum spatial resolution can be achieved with the sample as close as possible to the detector (Lehmann *et al.*, 2007 ▸), equivalent to Pos. A in Fig. 1 ▸. Unfortunately, there is a trade-off between the highest possible spatial resolution and the quantification of the transmitted signal with state-of-the-art neutron instruments. This is because the closer the detector is to the sample, the larger is the angle of the scattering cone covered by the detector and in particular by the sample projection (Fig. 1 ▸).

**Figure 2:**
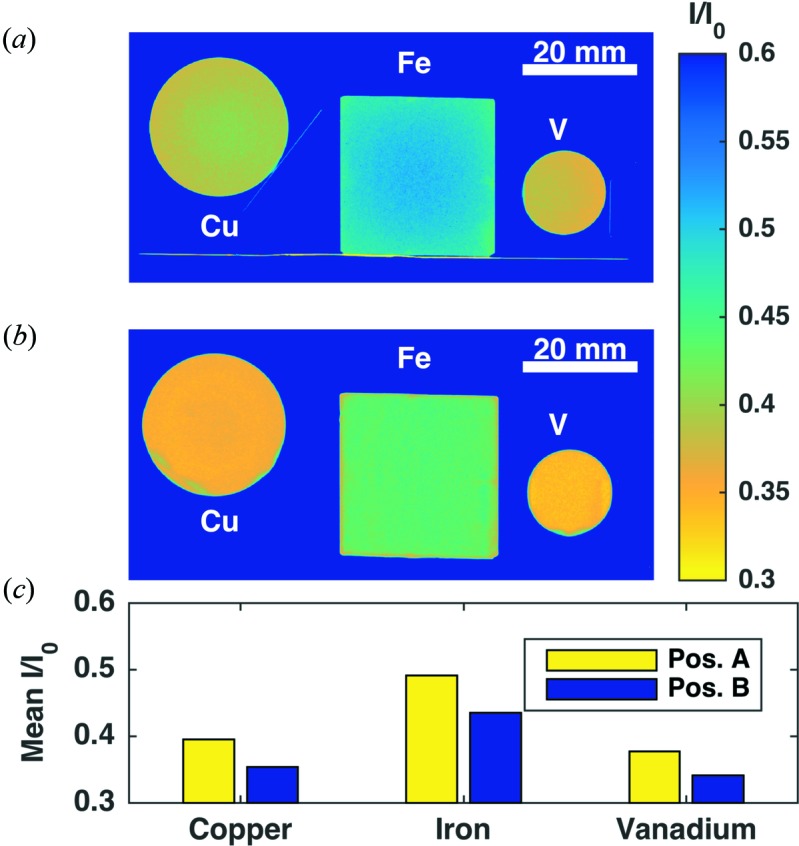
(*a*) Transmission images of Cu, Fe and V in Pos. A (*cf.* Fig. 1 ▸). (*b*) Transmission images of Cu, Fe and V in Pos. B. (*c*) Bar plot with the measured intensity averaged over the whole samples. All samples have 10 mm thickness. The increase in measured intensity for Pos. A with respect to Pos. B for Cu, Fe and V is 11.5, 12.9 and 10.6%, respectively. Samples were measured at the ICON beamline. All the images have been open-beam corrected.

Fig. 2 ▸ shows the results of radiography experiments performed at ICON. We choose three different sample shapes to show that the difference in transmission signal (from Pos. A and Pos. B) is predominantly not geometry dependent. The samples are a Cu cylinder with 25 mm diameter and 10 mm thickness, an Fe slab of 28 × 28 × 10 mm, and a V cylinder with 15 mm diameter and 10 mm thickness.

The detection system is composed of a ^6^LiF–ZnS scintillator with 200 µm thickness in combination with an Andor NEO sCMOS camera with 2560 × 2160 pixels and a 50 mm lens. The field of view was 112 × 94 mm, resulting in a pixel size of 44 µm. The samples were measured with an exposure time of 15 s. All the images presented in Fig. 2 ▸ have been dark-current and open-beam corrected.

The results of these experiments show the difficulties in correctly measuring polycrystalline structures, even with simple geometries. Figs. 2 ▸(*a*) and 2 ▸(*b*) show transmission images of Cu, Fe and V with samples at Pos. A and Pos. B, respectively. The pixel intensities are scaled between 0.3 and 0.6 for a better visualization of the scattering influence. A quantitative comparison of the mean transmission values is made in Fig. 2 ▸(*c*), showing a deviation larger than 10% between the two measurements. The details of polycrystalline sample scattering, and in particular how it affects neutron imaging, will therefore be introduced to address these challenges. In §2 we describe the underlying physics and neutron instrument parameters which constitute the basis for the Monte Carlo model. Subsequently, we describe in detail how the model is built and what it is capable of. Finally, a validation of the model by comparison with radiography and tomography experiments and a discussion of the results are given.

## 2. Theory   

### 2.1. Scattering cross sections   

Polycrystalline materials have an ordered periodic structure at the atomic level, which allows diffraction techniques to study their properties. Correspondingly, in neutron transmission experiments the structure of polycrystals should be taken into consideration if one aims to quantify the attenuation of the sample. To show the importance of these considerations, this work compares the accuracy of attenuation coefficients calculated with the hypotheses of amorphous material and structured material. The web site of the National Institute of Standards and Technology includes the *Neutron activation and scattering calculator* (https://www.ncnr.nist.gov/resources/activation/) tool, which offers a quick way of evaluating the attenuation of amorphous materials for a monoenergetic neutron beam. The calculation of the neutron attenuation coefficient for this tool follows 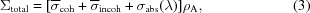
where 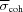 and 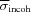 are the coherent and incoherent cross sections of the nucleus, respectively, 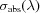 is the absorption cross section, and 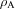 is the atomic density. In equation (3) we observe the wavelength dependence of the absorption cross section, whereas the total coherent and incoherent scattering contributions to the attenuation coefficient are assumed to be constant. The cross sections are defined as 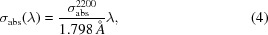


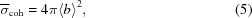


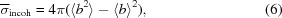
where λ is the neutron wavelength, 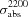 is the absorption cross section for thermal neutrons at a wavelength of 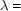 1.789 Å (equivalent to a neutron speed of 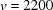
 m s^−1^), and *b* is the scattering length of the nucleus.

The calculation of the attenuation coefficient taking into account the structure of the material is 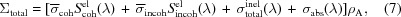
where the scattering functions *S* describe the influence of the spatial arrangement of the nuclei and their corresponding dependence on the neutron wavelength and 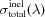 is the total inelastic scattering cross section. The first component of equation (7) accounts for the coherent elastic component of the scattering cross section and is calculated by means of the structure factor 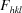 and the interplanar distance 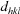 for every set of planes in the crystal (Fermi & Marshall, 1947 ▸):
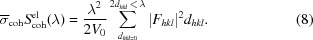
where *V*_0_ is the unit-cell volume. Note that this equation assumes the crystal to be a powder-like assembly of small crystal grains with random orientation. If this assumption does not apply, for example because of texture, orientation-dependent weighting factors have to be added (Woracek *et al.*, 2017 ▸). The scattering function 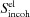 of equation (7) accounts for the elastic contribution of the incoherent scattering, based on the assumption of the thermal motion of the nuclei (Debye, 1913 ▸; Waller, 1923 ▸). The complete formulation of 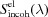 and 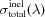 is given by Granada (1984 ▸) and Vogel (2000 ▸). It was later implemented by Boin (2012 ▸) in the *nxs* program library for cross section calculations, which we use in this work. In this model, the coherent and incoherent scattering, elastic and inelastic scattering, and absorption are included, taking into account the crystalline structure of the material and the phonon contribution assuming a powder polycrystal. A March–Dollase model for texture characterization is included in the *nxs* program library, although it has not been used in this work. Magnetic interactions are not considered.

### 2.2. Input parameters of the model   

**Figure 3:**
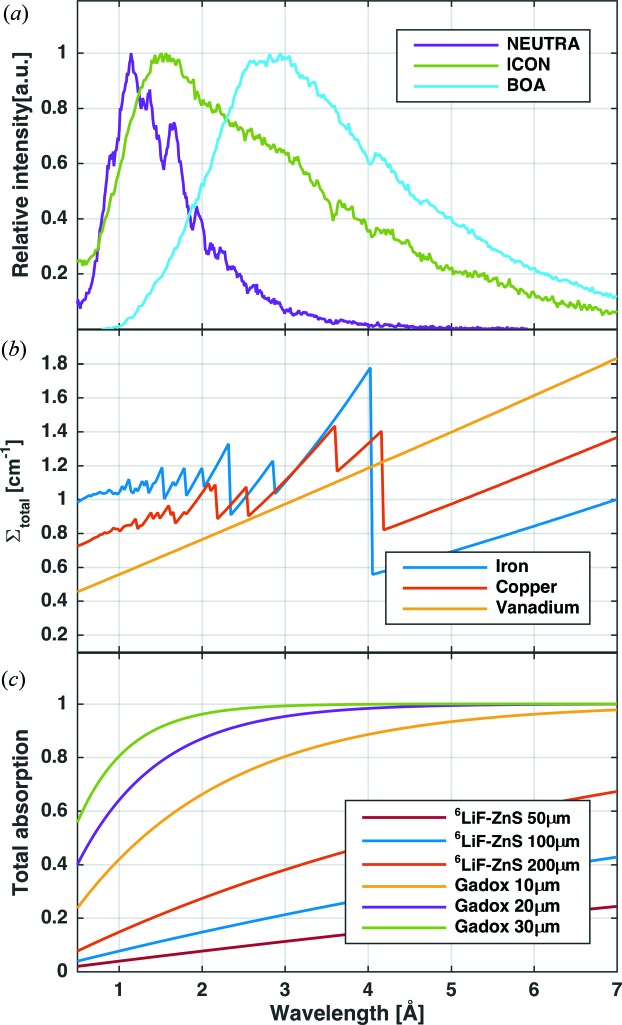
(*a*) Wavelength spectra of three imaging beamlines at PSI: NEUTRA (thermal), ICON (thermal–cold), BOA (cold). (*b*) Attenuation coefficients for Fe, Cu (with Bragg edges) and V computed with *nxsPlotter*. (*c*) Neutron absorption probability of different scintillator compositions and thicknesses.

The *nxs* library makes the neutron cross section calculation available for Monte Carlo simulations and as a cross section plotting tool: the *nxsPlotter*. In the following, a Monte Carlo simulation for neutron imaging experiments has been realized, paying attention to the most important influences on the measured and simulated results. These are visualized in Fig. 3 ▸: (*a*) the incident neutron spectrum, (*b*) the sample–neutron interaction and (*c*) the absorption probability of the scintillator screen. Notice that this model requires the wavelength spectrum of the beamline.

The curves for the different scintillators have been calculated from the thickness of the screen, from the binder–scintillator ratio and by considering a linear behaviour of the absorption cross section (either ^6^Li or natural Gd) with respect to the thermal wavelength (Table 1 ▸).

**Figure 4:**
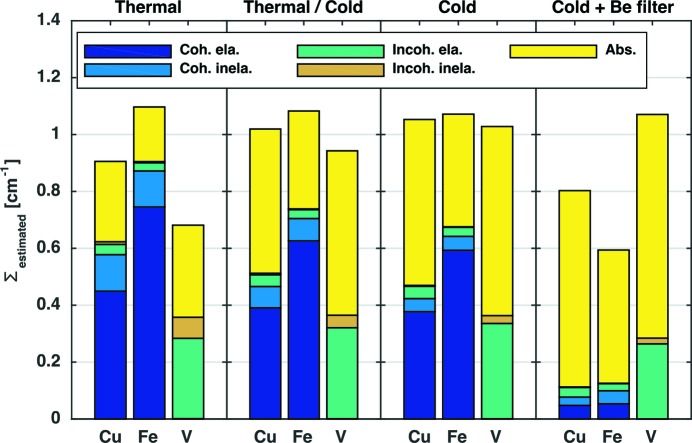
Attenuation coefficients for Fe, Cu and V computed using *nxsPlotter* data and the spectra from the different beamlines. They are split into the following contributions: coherent elastic, coherent inelastic, incoherent elastic, incoherent inelastic and absorption. The bar diagrams show the behaviour of Fe, Cu and V in a thermal spectrum (NEUTRA), thermal and cold (ICON), cold (BOA), and cold with beryllium (Be) filter. The Be filters out neutrons below 4 Å, thus leaving only the cold tail of the neutron spectrum and greatly reducing the effect of Bragg scattering.

Using the data in Fig. 3 ▸, an estimation of the attenuation coefficient value for white beam neutron imaging can be obtained, by calculating the mean of the attenuation coefficient weighted by the neutron spectrum:
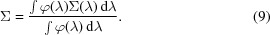
For a better understanding of the different attenuation contributions, Fig. 4 ▸ shows the individual components of the macroscopic cross section calculated for Cu, Fe and V on different beamlines. The different contributions have been calculated using the *nxsPlotter* [which is based on equation (7)] and the beamline spectra to obtain the mean of the cross sections weighted [equation (9)] by the neutron spectrum.

Fig. 4 ▸ shows the relevance of the different scattering mechanisms for Fe, Cu and V on beamlines with different spectra. Because V has a very small coherent cross section value, the coherent cross section does not contribute significantly to the total attenuation coefficient. For that reason, no Bragg edges are seen in the attenuation spectrum of V (Fig. 3 ▸ *b*). For coherent scatterers (Fe and Cu), neutrons with wavelength longer than 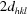 cannot be scattered coherently and elastically by the crystal lattice, so the attenuation coefficient for both Fe and Cu drops shortly after 4 Å (Figs. 3 ▸ *b* and 4 ▸). Fig. 4 ▸ also illustrates why it is important to take the wavelength-dependent coherent cross section into account if one aims to quantify the transmitted signal. On a thermal beamline, Fe is the most attenuating, followed by Cu and then V. On a cold beamline, all three have similar attenuation values. Finally, when the Be filter is used, and hence only neutrons with wavelengths larger than 4 Å contribute, the order is reversed (V is the most attenuating, then Cu and then Fe). The Be filter unit is normally placed between the pinhole and the sample and removes from the beam neutrons with wavelength shorter than 4 Å. In the following we introduce the beamline parameters, the *nxs* library and the detector behaviour into the Monte Carlo model.

## 3. The Monte Carlo model   

The Monte Carlo model has been coded using *McStas 2.a* (Lefmann & Nielsen, 1999 ▸), a neutron ray-tracing package used to simulate neutron instruments and experiments. The dimensions of the beamline and the position and geometry of different components are those depicted in Fig. 1 ▸. They are represented by virtual components, many of them available as generic modules in the *McStas* library. The moderator surface is a *Source_gen* component which uses the spectral data of the different beamlines as input. The pinhole is a circular *Slit* component. The sample is a *Sample_nxs* component, which follows the equations defined in §2 and includes the crystallographic properties of the samples. The detector is represented by a modified position sensitive detector (PSD) which weights the value of every neutron depending on the probability of being absorbed by the scintillator, following the curves of Fig. 3 ▸(*c*).

**Figure 5:**
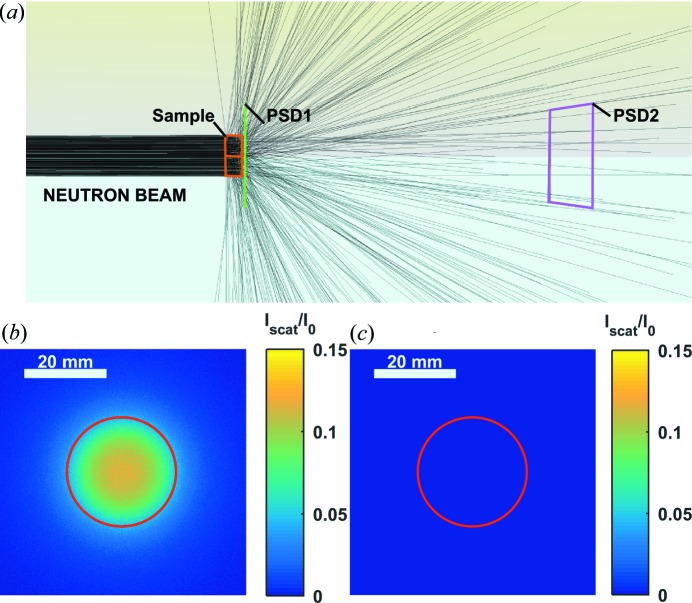
(*a*) *McStas* simulation of 1000 neutrons scattering from a Cu cylinder of 25 mm diameter and 10 mm thickness. (*b*) Intensity of the scattered neutrons detected by PSD1 in contact with the sample with respect to the open beam. (*c*) Intensity of the scattered neutrons detected by PSD2 at 200 mm from the sample with respect to the open beam. PSD1 and PSD2 are 200 mm apart, like Pos. A and Pos. B in Fig. 1 ▸, but in this case the detector is displaced, not the sample.

*Sample_nxs* is the implementation of the *nxs* program library into a *McStas* component. The code allows the user to choose between a cylindrical, slab or three-dimensional geometry from a given file. To illustrate this description, Fig. 5 ▸ shows a three-dimensional snapshot of 1000 neutrons scattering from a Cu sample, together with the relative scattering intensity measured with a PSD in close contact with the sample and another one 200 mm downstream. The number of neutrons in Fig. 5 ▸ was chosen in order to provide a good visualization of the scattering phenomenon; therefore only 1000 neutrons are shown.

The sample-to-detector distance has a large influence on the fraction of scattered neutrons detected. The smaller the distance, the larger the scattering angle range covered by the detector, but in particular also by the projection of the sample itself. By modifying the PSD monitor component in *McStas*, one can obtain data about the incident angle at which scattered neutrons enter the detector during the measurement. The measured dimension is defined as 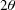 in Fig. 1 ▸, and it can be estimated from the simulation for the three different samples of study.

**Figure 6:**
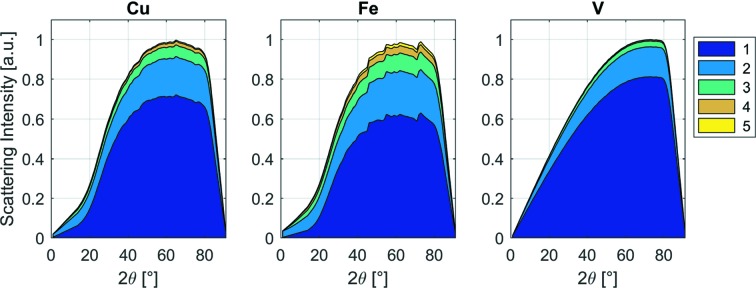
Scattering intensity at the imaging plane coming from the sample as a function of 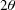 from white beam radiography simulations with the NEUTRA spectrum (Fig. 3 ▸) for Fe, Cu and V in position A. Notice the peaks arising from coherent scattering in Fe and Cu are not present in the scattering from V.

Fig. 6 ▸ shows how the scattering from the sample detected in the transmission detector has bumps in the case of Fe and Cu which are not present for V. These can be attributed to Debye–Scherrer rings (Cullity & Weymouth, 1957 ▸) arising from the coherent scattering in Fe and Cu, which are, despite the spatial smearing, still visible as bumps of intensity for certain scattering angles in the transmission detector. Note that the histogram integrates neutrons detected across the whole surface of the detector, which is 30 × 30 mm, and shows them as a function of 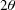 (Fig. 1 ▸). Transmitted neutrons are not shown in Fig. 6 ▸. In the next section a comparison of the experimental results and the Monte Carlo simulations is presented in order to validate the accuracy of the model.

## 4. Model validation and discussion   

In order to validate the Monte Carlo model, we compare the results of the simulations with experimental results for the scattering distribution and the tabulated attenuation coefficients of the materials, which do not yet account for the crystal structure. We present radiography and tomography results obtained at the NEUTRA beamline, *i.e.* utilizing a thermal neutron spectrum.

### 4.1. Scattering from the sample in neutron radiography   

**Figure 7:**
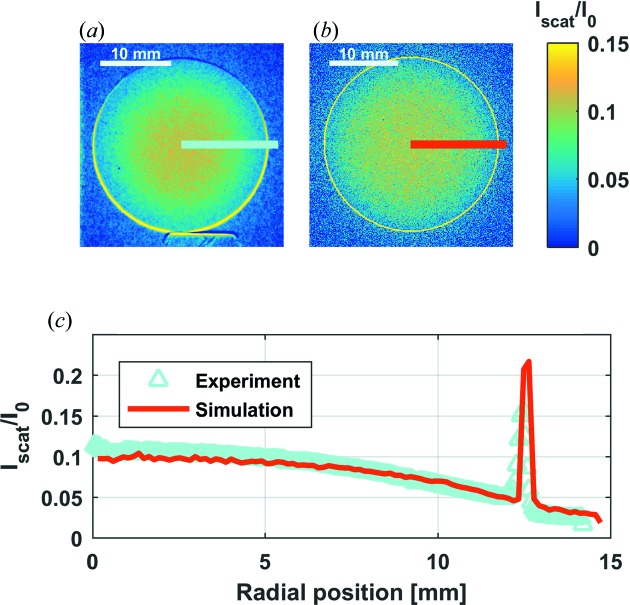
(*a*) Subtraction of a transmission image of the Cu sample at NEUTRA in Pos. B from another in Pos. A. (*b*) Subtraction of a simulated radiographic image of the Cu sample at NEUTRA in Pos. B from another in Pos. A. (*c*) Radial average of the intensities from experiments and simulations. Pos. A and Pos. B.

While performing neutron imaging experiments, we cannot avoid detecting the transmitted neutrons like we do in a simulation, but we can subtract the images acquired in Pos. A from those in Pos. B (Fig. 1 ▸) (Raventós *et al.*, 2017 ▸). By doing so, we can obtain qualitative information on the partition of neutrons scattered into the additional solid angle covered in Pos. B. Fig. 7 ▸ shows a comparison of the scattered contribution with the equivalent *McStas* simulation.

**Figure 8:**
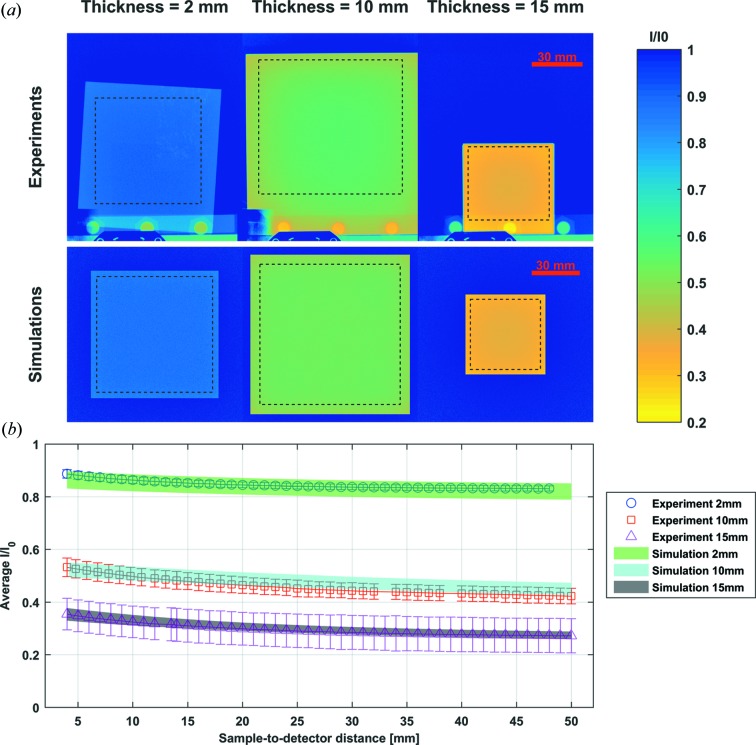
(*a*) Experimental and simulated radiographs of three Cu samples with different thicknesses at a sample-to-detector distance of 4 mm. (*b*) Normalized intensities averaged over the samples’ indicated region of interest at different sample-to-detector distances.

Fig. 7 ▸ confirms how standard radiographic images of crystalline materials can be affected by scattering contributions, in this case up to 10% of the incident beam. The simulation is performed with 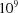 neutrons at Pos. A and Pos. B. The model can predict accurately the relative intensity of the scattering at any given sample-to-detector distance, as can be seen in Fig. 8 ▸.

Fig. 8 ▸ shows radiographs of three Cu slabs with dimensions 80 × 80 × 2 mm, 100 × 100 × 10 mm and 50 × 50 × 15 mm, measured at the NEUTRA beamline for sample-to-detector distances between 4 and 50 mm. One can observe how for each of the three curves there is a visible decrease in measured intensity with increasing sample-to-detector distance, most notably in the first 15 mm.

The simulation results from Figs. 7 ▸ and 8 ▸ are in good agreement with the experimental results, underlining that our model assumptions and descriptions are sufficient for this kind of simulation.

### 4.2. Reference attenuation coefficients in neutron tomography   

Subsequently, tomography was performed on the three samples at the NEUTRA beamline with the detector in Pos. B. The detector in this case was what we refer to as the Midi-Box camera setup: a ^6^LiF–ZnS scintillator with 100 µm thickness, and an Andor NEO camera with sCMOS sensor and 2560 × 160 pixels using a Nikkor 50 mm *f*/1.4. The camera was placed at a distance from the scintillator that provided a field of view of 148 × 176 mm and a pixel size of 69 µm. The three samples were measured simultaneously by using the POLYTOM device (Trtik *et al.*, 2016 ▸), which translates the movement of a single rotation axis to three different rotation stages enabling multiple simultaneous tomographic scans. The exposure time was 15 s per projection. The tomograms were reconstructed from 1125 projections over 360°, with the commercial software *octopus 8.9.1* (https://octopusimaging.eu/). Before reconstruction, standard corrections for dark-current and open-beam normalization were performed. Data were exported as single-precision floating-point slices to evaluate the attenuation coefficients measured in the bulk of the samples.

In order to simulate the tomograms each material was exposed to 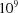 neutrons for each of the 360 radiographs over 360° used for the simulated tomography reconstructions, with a total simulation time of 15 h per tomographic scan. However, because of computational time constraints, a Maxwellian approximation of the NEUTRA spectrum was used for the simulated tomography, instead of the experimental NEUTRA spectrum. This way, the computation time can be decreased 60-fold, and tomographic simulations can be performed in a reasonable time.

**Figure 9:**
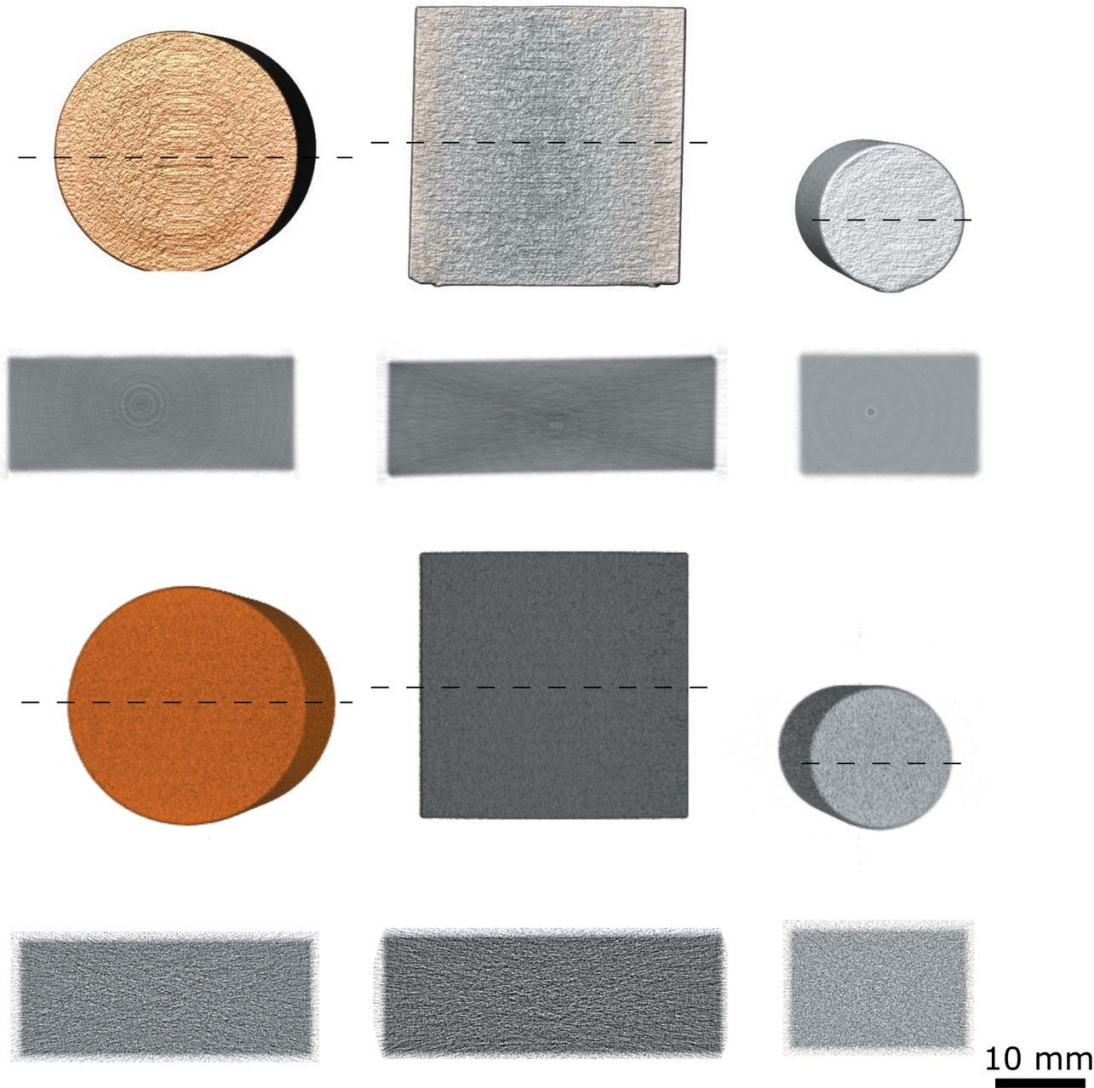
Three-dimensional reconstruction of the Cu, Fe and V tomography from the experiment (top) and the simulation (bottom). The dotted lines represent the section plane from which the slices underneath have been extracted and displayed with the same greyscale. The rotation axis for all cases is vertical with respect to the three-dimensional volumes shown in this figure.

One can observe in Fig. 9 ▸ how the experimental tomograms show some reconstruction artefacts such as rings and streaks, probably due to the experimental conditions. On the other hand, the simulated tomograms show a higher variability of the voxel value owing to the poor statistics from the computational constraints, while the rotation axis was known in advance and no ring artefacts seem to appear. Nevertheless, both show comparable values for the attenuation in all three materials.

For the quantitative comparison of the attenuation coefficients, the NIST calculator value was obtained from the http://www.ncnr.nist.gov/resources/activation/ using a thickness of 10 mm, a neutron wavelength of 1.798 Å and the nominal values for density, which are 7.87 g cm^−3^ for Fe, 8.96 g cm^−3^ for Cu and 6.11 g cm^−3^ for V.

The values of the attenuation coefficients using the *nxsPlotter* without simulations were calculated following the same steps as in Fig. 4 ▸. In this case, the wavelength-dependent absorption curve of the 100 µm ^6^LiF–ZnS thickness scintillator is weighted in the calculation.

**Figure 10:**
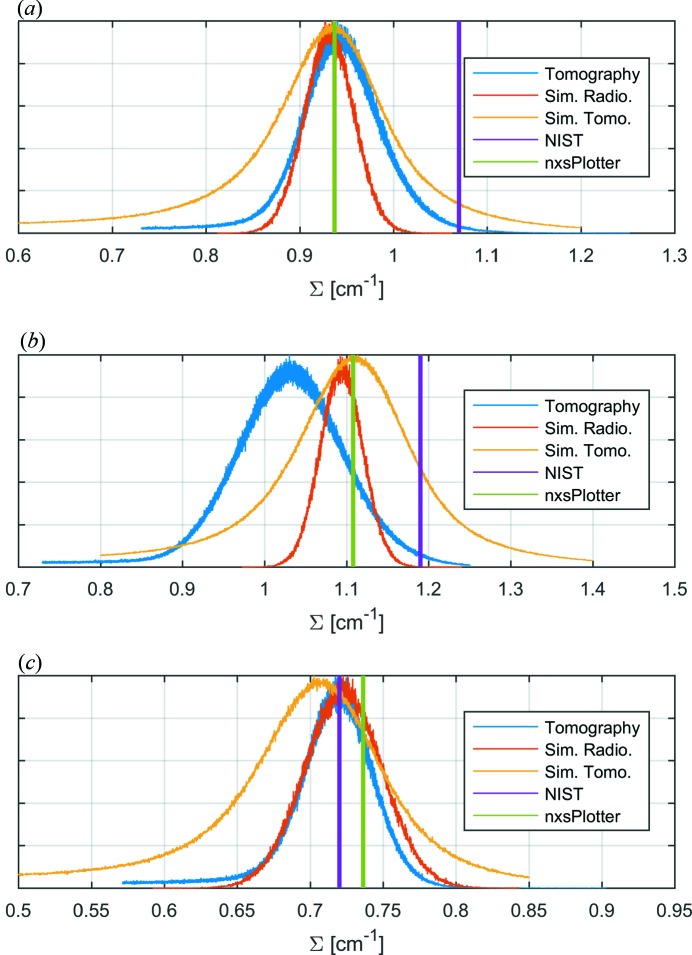
Comparison of the attenuation coefficients obtained by tomography, simulated radiography, simulated tomography, NIST and *nxsPlotter* for Cu (*a*), Fe (*b*) and V (*c*). For all three graphs the tomography and simulation distribution values are normalized to the most frequent value.

The simulation results were obtained from the simulated tomographic reconstruction using the approximated Maxwellian spectrum source and with simulated radiographs using the experimental spectrum source. The latter were optimized to reduce the statistical uncertainty by simulating samples with a large surface and a constant thickness of 10 mm (simulation time 18 h per radiograph). These simulation values of the attenuation coefficient are compared with the experimental values, those provided by the NIST calculator and those calculated with the *nxsPlotter* in Fig. 10 ▸.

Naturally, in contrast to the tabulated values, the measured and simulated values display a distribution due to statistical uncertainty and are presented as such in Fig. 10 ▸.

The mean values of the attenuation coefficient obtained by tomography are 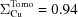
 cm^−1^, 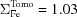
 cm^−1^ and 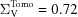
 cm^−1^. The spread of the resulting values in terms of full width at half-maximum (FWHM) is 0.12 for Fe, 0.09 for Cu and 0.04 for V. Differences in FWHM of the attenuation coefficient among samples are due to variations in the dimensions and attenuation coefficient of the Fe, Cu and V samples.

The mean values of the attenuation coefficient obtained by simulated radiography are 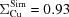
 cm^−1^, 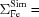 1.09 cm^−1^ and 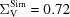
 cm^−1^. The FWHM for the simulated data is 0.06 for Fe, 0.06 for Cu and 0.06 for V. As can be seen, here the values of the attenuation coefficient have little impact on the uncertainty of the measurement, while they differ from the experimental results where some projections are more affected than others owing to the shape and dimensions of the sample and the corresponding projections.

The mean values of the attenuation coefficient obtained by simulated tomography are 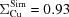
 cm^−1^, 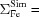 1.11 cm^−1^ and 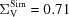
 cm^−1^. The FWHM for the simulated data is 0.16 for Fe, 0.13 for Cu and 0.18 for V. Given the spread of the distributions and the fact that they were performed with a Maxwellian approximation of the spectrum, we do not recommend this method for the estimation of the attenuation coefficients.

The values of the attenuation coefficient obtained using the NIST database are 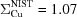
 cm^−1^, 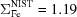
 cm^−1^ and 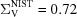
 cm^−1^.

The values of the attenuation coefficient obtained using *nxsPlotter* are 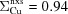
 cm^−1^, 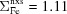
 cm^−1^ and 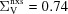
 cm^−1^. The results of *nxsPlotter* are interpreted as the value of the attenuation coefficient that one would obtain from the simulation if the sample were infinitely thin. Since the samples’ dimensions are in the range of tens of millimetres, the simulated and experimental attenuation coefficients are affected by beam hardening, which is not accounted for in the *nxsPlotter* calculation. Therefore, the *nxsPlotter* value will always give a slightly higher attenuation coefficient value than the simulation value.

As can be seen, the nuclear nominal values are 13.8% higher for attenuation in the case of Cu and 15.5% higher in the case of Fe when compared with the mean value obtained from tomography. For the simulated radiography, the deviation is 1.1% in the case of Cu and 5.8% in the case of Fe between mean values. Differences in attenuation values between the Fe experiment and the simulation are attributed to the Fe sample being strongly textured. Still, the results show improvement as reference value when compared to the nuclear nominal values. If known, texture can be accounted for in the Monte Carlo model with a March–Dollase factor, which can be enabled in *Sample_nxs*.

In the case of V, where coherent elastic scattering and hence the crystal structure has no impact, the tomography, simulated radiography, NIST and *nxsPlotter* values coincide well.

## 5. Conclusions   

Discrepancies between neutron imaging measurements of standard polycrystalline materials at different sample-to-detector distances motivated the creation of a Monte Carlo simulation tool. The model, which includes beamline parameters, crystallographic information on the samples, and scintillator composition and thickness, could reproduce the correlation between distance and measured deviations on the basis of scattering effects. When comparing the results from experiments with simulations, the model appears in good quantitative agreement with the measurements. Comparing attenuation coefficients measured in the volume in tomography experiments with standard nuclear values often used as reference values and the Monte Carlo simulations, the model-based simulation provides values which are more precise for strong coherent scattering materials like Fe and Cu. For a material without a relevant coherent elastic scattering contribution like V, on the other hand, the reference value not accounting for crystalline structure appears just as precise as a detailed simulation. The examples underline that for a structural material like Cu, quantification without accounting for the crystalline nature of the material and the corresponding scattering effects can introduce errors significantly larger than 10% in quantification.

As has been shown, if one aims to quantify the transmitted signal of a Cu sample in close contact with the scintillator and using the amorphous hypothesis, one will be not only adding up to 10% of the open-beam signal to the transmission signal in the form of scattered neutrons, but also using an attenuation coefficient value as reference which is 13.8% higher than the real one.

This model has the potential to become a routine modelling tool for neutron radiography measurements. The strength of our approach lies in the flexibility of the model with respect to beamline parameters and detector types, being able to assess neutron imaging experiments for any instrument and detector configuration. All previously mentioned custom *McStas* component are now available in the *McStas-Imaging-Tools* GitHub repository (https://doi.org/10.5281/zenodo.1041731).

## Figures and Tables

**Table 1:** Scintillator composition and total neutron absorption at 1.8 Å

	Air (µm)	Binder (µm)	ZnS (µm)	^6^LiF/Gadox (µm)	Absorption at 1.8 Å
^6^LiF–ZnS 50 µm	10.5	12	15.5	12	0.07
^6^LiF–ZnS 100 µm	21	24	31	24	0.13
^6^LiF–ZnS 200 µm	42	48	62	48	0.25
Gadox 10 µm	1	0.6	–	8.4	0.62
Gadox 20 µm	2	1.2	–	16.9	0.86
Gadox 30 µm	3	1.8	–	25.3	0.95
